# Research on the Soft-Sensing Method of Indicator Diagram of Beam Pumping Unit

**DOI:** 10.3390/s24061794

**Published:** 2024-03-11

**Authors:** Huaijun Zhao, Junping Wang, Tianyu Liu, Yang Yu, Dingxing Hu, Chenxin Cai

**Affiliations:** 1 Faculty of Mechanical and Precision Instrument Engineering, Xi’an University of Technology, Xi’an 710048, China; xaut_zhj@139.com (H.Z.);; 2 Oil & Gas Technology Research Institute, Changqing Oilfield Company, PetroChina, Xi’an 710018, China; 3 Xi’an Hi-Rate Electrical Power Technology Co., Ltd., Xi’an 710018, China

**Keywords:** indicator diagram, soft measurement technology, beam pumping unit, electric parameter method, petroleum

## Abstract

An accurate calculation of the indicator diagram of a pumping unit is the key factor in analyzing the performance of an oilfield production and operation and in preparing and optimizing an oilfield development plan. Aiming at the problems of the poor stability of the conventional load-displacement sensor method and the wave equation method, owing to the influence of an alternating load on the force sensor and the difficulty in measuring the crank angle using the electrical parameter method, a new soft sensing method employing the input electrical parameters of the motor and the beam inclination has been proposed to obtain the indicator diagram. At first, this method is established based on the beam angle of the pumping unit, which is easily measured using the suspension point displacement mathematics calculation model and the torque factor. Subsequently, the electric motor input parameters, the parameters of the four-bar linkage, and the relationship between the polished rod load have been established. Finally, the motor and the beam angle of the measured electrical parameters have been substituted into the calculation of the suspension point displacement and load value and pull in accordance with the guidelines to eliminate the singularity mutation values. After processing the measured data through a Butterworth filter, the indicator diagram is obtained. The results of the engineering experiment and application show that the average relative error of the method is less than 3.95%, and the maximum relative error remains within 2% for 6 months, which verifies the stability of the soft sensing method.

## 1. Introduction

The indicator diagram of a beam pumping unit is a two-dimensional closed curve mapped by the relationship between the polished rod load and the displacement in a single stroke cycle, which carries important working condition information of the pumping unit system and well pump operation. Real-time and accurate designing of an indicator diagram is a key factor for analyzing the oilfield production and operation dynamics, formulating and optimizing the oilfield development plans [1,2,3], and is also one of the research hotspots in terms of its mechanical production and efficiency improvement methods owing to the development trend of improving the quality and efficiency in the oilfield industry. 

At present, there are three main methods to obtain the indicator diagrams of conventional beam pumping units. The first is the load-displacement sensor method. A load sensor is used to measure the real-time load of the polished rod of the pumping unit, and linear or angular displacement sensors, such as various indicators installed on the polished rod and the beam of the pumping unit, are used to measure the real-time displacement of the polished rod to directly obtain the indicator diagram [4]. Because this method is simple and direct, it still occupies the leading position in the market today. However, its disadvantage is that its force sensor bears the impact of alternating load stretching for a long time. In addition, it is prone to zero drift, has poor stability, and has a short life [5]. The second method of obtaining the indicator diagram is the wave equation method, in which the vibration of the sucker rod and oil column is received in the form of a stress wave, and the pump indicator diagram is calculated using the wave equation. For example, Gibbs and Neeley established a single-stage damped wave equation of vibration by analyzing the longitudinal motion of the polished rod of a pumping unit [6], Doty and Schmidt added the oil column vibration analysis following Gibbs and Neeley’s research and established a two-dimensional calculation model [7]. Y. Peng et al. combined the matrix recursion and trigonometric series recursion techniques to establish a three-dimensional mathematical model, which improved the measurement accuracy of this method [8]. However, such methods involve a large amount of calculation in solving differential equations. In addition, their force sensors are also subject to long-term alternating load tension, which can easily lead to aging and poor stability. The third method for obtaining the indicator diagram is the indirect method, in which the measured electrical parameters of the motor or the downhole pump condition mechanism model are used. For example, Li et al. obtained the indicator diagram based on the method of calculating the load torque according to the motor direct torque control theory and the power transmission process of the pumping machine [9]. Lv et al. established the indicator diagram solution model based on electrical parameters by studying the change in the relationship between the efficiency and torque transmission of the motor, belt, and reducer [10]. Li et al. predicted the indicator diagram by establishing the mechanism model of the oil, gas, and water multiphase fluid pumping process [11]. However, the torque factor calculation involved in this method is generally based on the crank angle, which is unlikely to play an efficient role in engineering applications due to the difficulty in its installation mode, inconvenience of maintenance, and other factors of the crank angle measurement sensor or the complex downhole working conditions.

**Figure 1:**
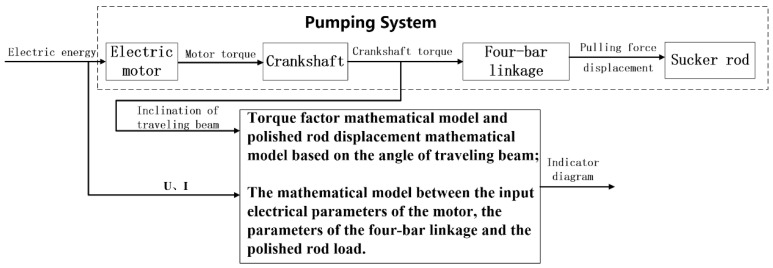
Soft-sensing technology roadmap of the pumping unit indicator diagram.

In this study, firstly, the mathematical models of displacement and torque factor of the horsehead suspension point of the pumping unit have been established based on the easily measured beam inclination angle. Secondly, by analyzing the motion law and the energy flow mechanism of the beam pumping unit system [12,13,14,15,16], the correlation model of the input electrical parameters of the motor, the parameters of the four-bar linkage mechanism of the pumping unit, and the pumping unit suspension point load has been established. The real-time collected beam inclination angle and input electrical parameters of the motor have been substituted into the above model, and the polished rod displacement and load value have been calculated. After further data processing and correction, an online soft measurement of the indicator diagram of the beam pumping unit has been realized. The specific technical route is shown in Figure 1. Where, U and I are respectively the input voltage and input current of the motor.

## 2. Establishment of the Mathematical Model for Measuring the Polished Rod Displacement of the Pumping Unit

**Figure 2:**
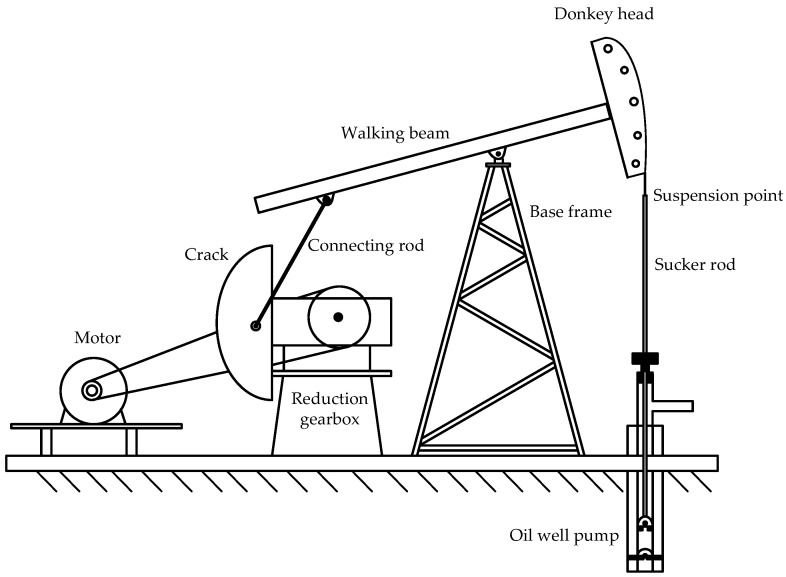
Structural diagram of beam pumping unit.

The polished rod displacement is the running track of the horsehead polished rod of the pumping unit moving between the upper and lower dead points. In order to obtain the real-time value of the suspension displacement, it is necessary to establish the correlation between the inclination of the beam and the suspension displacement. The structural diagram of the beam pumping unit is shown in Figure 2.

**Figure 3:**
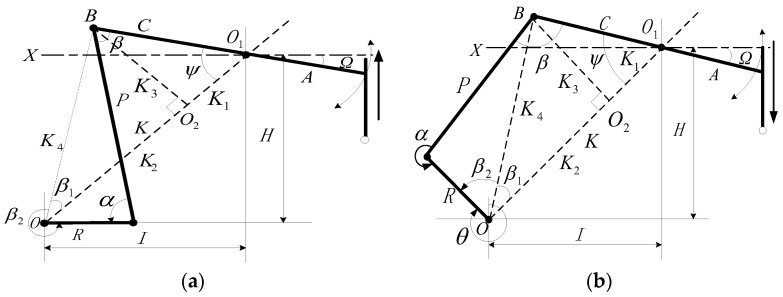
Structure diagram of a beam-type pumping unit for (**a**) on up-stroke and (**b**) on down-stroke.

Figure 3 shows a schematic of the four-bar linkage of the beam pumping unit. The crank is set to rotate clockwise. Here, *A* is the length of the front arm of the traveling beam, *C* is the length of the rear arm of the traveling beam, *R* is the length of the crank, *K* is the length of the base rod (also known as the pole distance, the distance from the center of the reducer output shaft to the center of the crank pin bearing), *P* is the length of the connecting rod (the distance from the center of the beam bearing to the center of the crank pin bearing), *H* is the vertical distance from the traveling beam support center to the center of the reducer output shaft, and *I* is the horizontal distance from the traveling beam support center to the center of the reducer output shaft. All the distance units are in m. Since different types of beam pumping units have different mechanical parameters, the distance values of the above parameters should be derived from the specific type of beam pumping unit. *Ω* is the inclination angle of the traveling beam (the angle between the traveling beam forearm and the horizontal line *X*), which is negative when the traveling beam forearm is below the horizontal line, and positive when the traveling beam forearm is above the horizontal line. The amplitude can be measured online by the inclination sensor installed at the traveling beam, and its unit of measurement is in degrees. *ψ* is the included angle between the rear arm of the traveling beam and the base rod (in degrees), *β* is the included angle between the rear arm of the traveling beam and the connecting rod, *β*_1_ is the included angle between *K*_4_ and *K*_2_, *β*_2_ is the included angle between *R* and *K*_4_ (in degrees), *θ* is the crank angle, (in degrees) obtained by setting the position of the crank at 12 o’clock as zero and measuring along the crank rotation direction, α is the included angle between the connecting rod and the crank, taken as a positive value in the counterclockwise direction (in degrees), *K*_1_, *K*_2_, *K*_3_, and *K*_4_ are the lengths of each dotted line (in m).

When the suspension point is at the bottom dead center limit position, the maximum included angle, *Ψ_b_*, between the rear arm of the traveling beam and the base bar is

(1) $$\psi_{b}=\arccos\left(\frac{C^{2}+K^{2}-{\left(P+R\right)}^{2}}{2CK}\right)$$

It is considered that the lower dead point is the zero point of the polished rod displacement and the upward direction is the positive direction. Thus, the polished rod displacement, *S_i_*, at any time is

(2) $$\begin{array}{l} S_{i}=\left(\Omega_{i}+\left(\psi_{b}-\arctan\frac{H}{I}\right)\right)\frac{\pi A}{180}\\ \;\;\;=\left(\Omega_{i}+\arccos\left(\frac{C^{2}+K^{2}-{\left(P+R\right)}^{2}}{2CK}\right)-\arctan\frac{H}{I}\right)\frac{\pi A}{180} \end{array}$$

In Equation (2), *Ω_i_* is the real-time sampling value of the beam inclination, the subscript *i* represents the *i* th sampling point in a stroke cycle, *i* = 1, 2, 3… The parameters *C*, *K*, *P*, *R*, *H*, *I*, and *A* are determined by the mechanical parameters of the pumping unit itself. As long as the inclination angle, *Ω_i_*, of the traveling beam is measured, the suspension displacement, *S_i_*, can be calculated.

**Figure 4:**
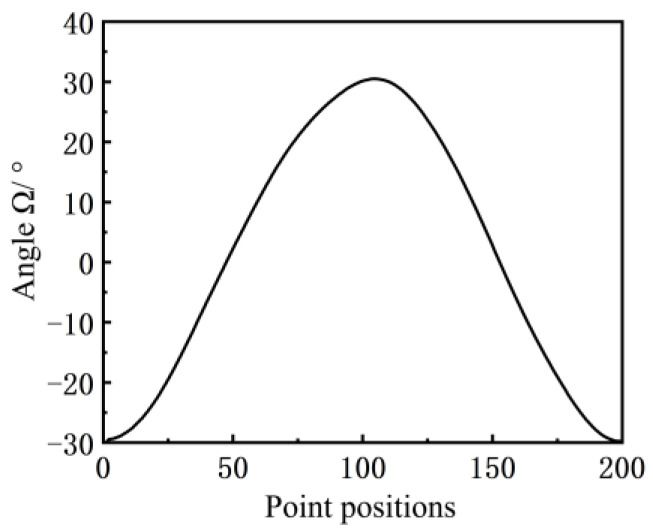
Inclination data of the beam.

**Figure 5:**
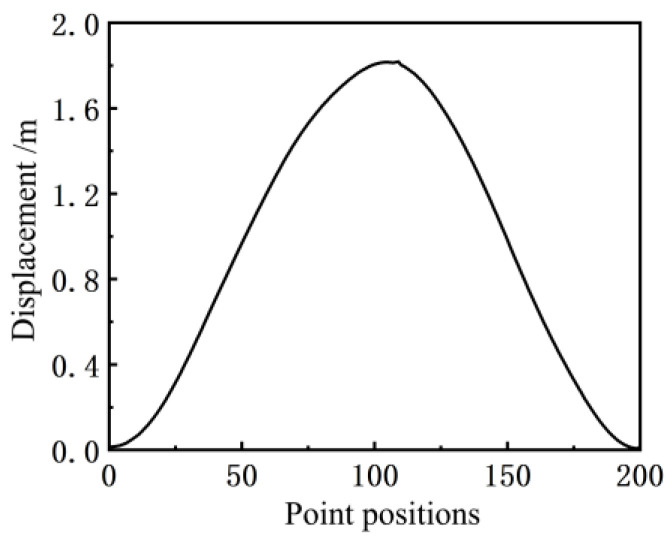
Displacement curve.

In Well Wang 11–251 in the Wangyao Operation Area of No. 1 Oil Production Plant of Changqing Oilfield, the inclination angle curve of the beam of the pumping unit (model CYJY5–1.8–13HB) was measured with the inclination sensor (model MSH527), as shown in Figure 4. The suspension point displacement curve obtained from the calculation and simulation using Equation (2) is shown in Figure 5.

## 3. Establishment of the Mathematical Model for the Soft Sensor of the Polished Rod Load

Through the motor of the pumping unit system, the input electrical energy is converted into the output torque of the motor, which is converted into the output torque through the transmission of the crankshaft. This is further converted into the vertical tension acting on the polished rod suspension point by the four-bar linkage to drive the pumping rod to move up and down. Based on the mechanism of energy flow, the correlation model between the input electrical parameters of the motor, the parameters of the four-bar linkage, and the polished rod load has been established, as presented in the subsection below, to realize an accurate measurement of the polished rod load.

### 3.1. Establishment of the Torque Factor Mathematical Model Based on the Angle of the Traveling Beam

The torque factor is the torque generated by the unit polished rod load on the crankshaft of the pumping unit. It is an important parameter connecting the polished rod load and the crankshaft torque. Its magnitude, $\bar{TF}$, is related to the geometric size of the pumping unit and the position of the crank, connecting the rod and the rear arm. It is expressed as

(3) $$\bar{TF}=\frac{A}{C}R\frac{\sin\alpha }{\sin\beta }$$

where *A*, *C*, *R*, *α*, and *β* are the same parameters as those defined in Figure 3. For a specific type of pumping unit, *A*, *C*, and *R* are determined. As long as, *α* and *β* at different values of, *θ* are obtained, for each crank angle can be calculated using Equation (3). This procedure is the current conventional calculation method.

However, in practical engineering applications, it is relatively difficult to install an angle-measuring device on the crank and its maintenance is inconvenient. In addition, there will be errors in the design, manufacturing, and installation of the pumping unit, which will affect the initial angle of the horsehead at the upper and lower dead points and will cause an error in identifying the upper and lower dead points of the horsehead as a function of *θ*. Therefore, in this study, the inclination angle, *Ω*, has been measured using the inclination sensor installed on the traveling beam to replace the conventional crank angle, *θ*. The mathematical model of the torque factor of the pumping unit has been established based on the inclination angle of the traveling beam, and the torque factor has been calculated by measuring.

Further analysis of Figure 3 gives the following formulae (Equations (4)–(7)):

(4) $$\left\{\begin{array}{l} K_{1}=C\cos\left(\arctan\frac{H}{I}-\Omega \right)\\ K_{2}=K-K_{1}\\ K_{3}=C\sin\left(\arctan\frac{H}{I}-\Omega \right)\\ K_{4}=\sqrt{C^{2}+K^{2}-2CK\cos\left(\arctan\frac{H}{I}-\Omega \right)} \end{array}\right.$$

It should be noted that in Equation (4), is a negative number according to the relevant provisions in Section 2.

(5) $$\beta_{1}=\arctan\left(\frac{K_{3}}{K_{2}}\right)$$

(6) $$\beta_{2}=\arccos\left(\frac{R^{2}+K_{4}^{2}-P^{2}}{2RK_{4}}\right)$$

(7) $$\sin\alpha =-\frac{K_{4}}{P}\sin\beta_{2}$$

During the up-stroke, as shown in Figure 3a, the four-bar linkage relationship gives

(8) $$\beta =2\pi +\alpha -\beta_{1}-\beta_{2}-\psi$$

During the down-stroke, as shown in Figure 3b, the four-bar linkage relationship gives

(9) $$\beta =\alpha -\beta_{1}-\beta_{2}-\psi$$

Comprehensive forms of Equations (8) and (9) can be taken from the periodicity of the sine function

(10) $$\beta =\alpha -\beta_{1}-\beta_{2}-\psi$$

Using the comprehensive forms of Equations (7) and (10), *α* and *β* can be expressed as

(11) $$\left\{\begin{array}{l} \alpha =-\mathrm{arc}\sin\left(\frac{K_{4}}{P}\sin\beta_{2}\right)\\ \beta =\alpha -\beta_{1}-\beta_{2}-\psi =\alpha -\beta_{1}-\beta_{2}+\Omega -\arctan\frac{H}{I} \end{array}\right.$$

Substituting Equations (11) into (3), we get

(12) $$\bar{TF}=\frac{A}{C}R\frac{\sin\alpha }{\sin\beta }=-\frac{A}{C}R\frac{\frac{K_{4}}{P}\sin\beta_{2}}{\sin\left(\alpha +\Omega -\beta_{1}-\beta_{2}-\arctan\frac{H}{I}\right)}$$

where *A*, *C*, *R*, *P*, *H*, and *I* are determined by the mechanical parameters of the pumping unit itself, and these parameters are determined and known for a specific pumping unit, whereas *α*, *β*_1_, *β*_2_, and *K*_4_ are functions of the above parameters and the angle of the traveling beam. Thus, the torque factor, $\bar{TF}$, is only a function of *Ω*. So far, we have established a torque factor calculation model of the pumping unit directly based on the angle, *Ω*, of the beam.

### 3.2. Establishment of the Correlation Model between the Crankshaft Torque and the Polished Rod Load of the Pumping Unit

The torque generated by the pumping unit at the crank output shaft will be affected by it’s balance mode. At present, there are three types of commonly used balancing methods: beam balance, crank balance, and composite balance. In this study, the beam pumping unit with crank balance has been taken as an example for the analysis. Other balance methods are similar.

**Figure 6:**
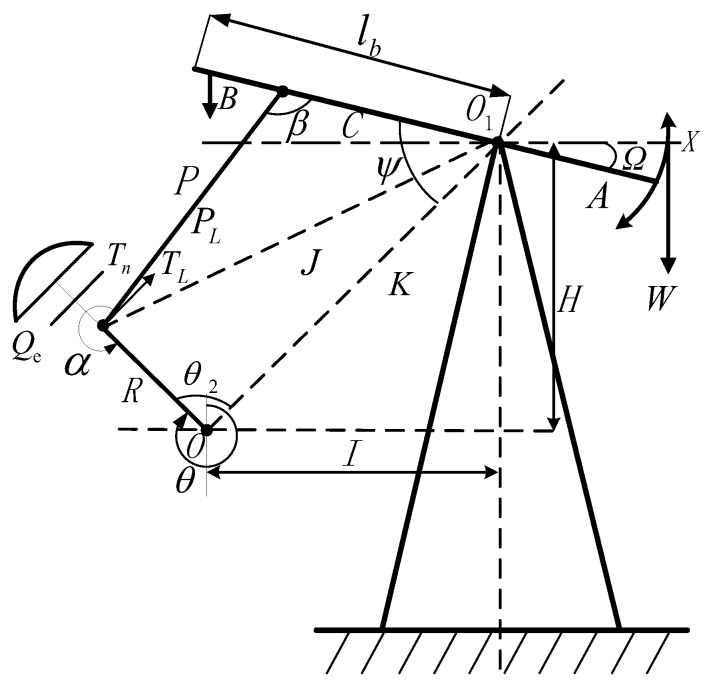
Force analysis diagram of a crank-balanced pumping unit.

Figure 6 shows the beam pumping unit with crank balance, where *W* is the polished rod load (in kN). The definitions of the other symbols are the same as in Section 2.

Consider the traveling beam as the research object. The connecting rod force, *P_L_*, can be obtained by taking the moment of all forces on the rotating center of the traveling beam as follows

(13) $$P_{L}=W\frac{A}{C}\frac{1}{\sin\beta }$$

Then, the component force, *T_L_*, of the connecting rod force, *P_L_*, in the tangential direction of the crank is

(14) $$T_{L}=-W\frac{A}{C}\frac{\sin\alpha }{\sin\beta }$$

Consider the crank as the research object. To lift the sucker rod string and oil column in the wellbore, the crankshaft output torque of the gearbox is *T_n_*. The torque generated by the gravity of the crank balance weight and the equivalent load, $Q_{e}$, of the gravity of the crank together overcome the torque generated by the tangential force, *T_L_* [17,18]. It is known from the crank balance condition that

(15) $$T_{n}+T_{L}R-Q_{e}R\sin\left(2\pi -\theta \right)$$

Then,

(16) $$\begin{array}{l} T_{n}=-T_{L}R-Q_{e}R\sin\theta \\ \;=\frac{A}{C}R\frac{\sin\alpha }{\sin\beta }\cdot W-Q_{e}R\sin\theta \\ \;=\bar{TF}\cdot W-Q_{e}R\sin\theta \end{array}$$

where $Q_{e}R\sin\theta$ in Equation (16) is the torque generated on the crankshaft by the self-weight of the crank and the crank counterweight, which is called the crank balancing torque.

By analyzing the four-bar linkage structure of the pumping unit in Figure 1, we get

(17) $$\theta =-\left(\beta_{1}+\beta_{2}-\arctan\frac{I}{H}\right)$$

The self-weight of the structural components, such as the beam, the horsehead, the connecting rod, and the beam of the pumping unit is not considered in Equation (16). When considering the structural imbalance of the pumping unit itself, in combination with Equation (17), Equation (16) can be further written as

(18) $$T_{n}=\left(W-B_{1}\cos\psi \right)\bar{TF}+Q_{e}R\sin\left(\beta_{1}+\beta_{2}-\arctan\frac{I}{H}\right)$$

where *B*_1_ is the structural unbalance weight of the pumping unit.

In order to facilitate engineering calculations, the influence of the beam swing angle can be ignored. Therefore, by simplifying Equation (18), we can get the correlation model of the crankshaft torque and the polished rod load of the crank-balanced beam pumping unit as shown in Equation (19) below:

(19) $$T_{n}=\bar{TF}\left(W-B\right)+Q_{e}R\sin\left(\beta_{1}+\beta_{2}-\arctan\frac{I}{H}\right)$$

where *B* is the structural unbalanced weight of the pumping unit, neglecting the swing angle of the beam.

### 3.3. Establishment of the Correlation Model between the Polished Rod Load and the Input Electrical Parameters of the Motor

In order to reduce the influence of the three-phase voltage asymmetry on the calculation of the output torque of the motor in the low-voltage power grid of an oil production well site, the positive sequence power, calculated using the symmetrical component method [19,20], has been used in this study as the motor input power.

Let the three-phase instantaneous voltage and current values of the motor input terminal be ($u_{a}$, $u_{b}$, $u_{c}$) and ($i_{a}$, $i_{b}$, $i_{c}$), respectively. The positive sequence voltage, $u_{+}$, current, $i_{+}$, and average positive sequence active power, $P_{1}$, are calculated using Equations (20)–(22), respectively, as follows:

(20) $$u_{+}=\frac{1}{3}\left(u_{a}+\alpha u_{b}+\alpha^{2}u_{c}\right)$$

(21) $$i_{+}=\frac{1}{3}\left(i_{a}+\alpha i_{b}+\alpha^{2}i_{c}\right)$$

(22) $$P_{1}=\int_{0}^{T}{u_{+}}^{T}i_{+}dt=3U_{+}I_{+}\cos\phi^{+}$$

where $\alpha =e^{j120^{\circ }}$, $\alpha^{2}=e^{-j120^{\circ }}$, α are unit vectors at 120° of the direction angle and $\phi^{+}$ are the power factor angles.

The relationship between the input power of the motor and the torque, $M_{ed}$ [21], of the crankshaft can be expressed as follows:

(23) $$M_{ed}=\frac{30P_{1}\eta \eta_{E}}{n\pi }$$

where η is the motor efficiency, dimensionless, n is the motor shaft speed, and $\eta_{E}$ is the transmission efficiency from the motor to the crankshaft. When the motor speed and the reducer output shaft speed are determined, *n*, η, $\eta_{E}$ are known quantities.

When the beam pumping unit is working, the torque generated by the polished rod load and the crank counterweight on the crankshaft (reducer output shaft) is balanced with the torque output to the crankshaft by the motor [22], i.e.,

(24) $$M_{ed}=T_{n}$$

In the comprehensive formulae given in Equations (19), (23) and (24), for the beam pumping unit with crank balance, the correlation between the polished rod load, *W*, and the input electrical parameters of the motor can be expressed as

(25) $$W=\frac{30P_{1}\eta \eta_{E}}{n\pi \bar{TF}}-\frac{Q_{e}R\sin(\beta_{1}+\beta_{2}-\arctan\frac{I}{H})}{\bar{TF}}+B$$

where *n*, η, $\eta_{E}$ are the inherent parameters of the motor. The weight of the crank balance and the equivalent load, $Q_{e}$, of the self-weight of the crank is relatively fixed and can be known by measuring once when the balance weight is adjusted. *R*, *H*, *I*, *B*, etc. are the mechanical parameters of the pumping unit. For a given model of the motor and the beam pumping unit, these parameters are known. The average active electric power, $P_{1}$, of the positive sequence input of the motor can be measured and calculated by the three-phase voltage and the three-phase current measurement module in real time. *β*_1_, *β*_2_, and $\bar{TF}$ are the only functions related to the inclination, Ω, of the traveling beam, Ω which can be measured in real-time by the inclination sensor of the traveling beam.

So far we have established a calculation model of the torque factor of the pumping unit directly based on the angle, Ω, of the beam, and a soft sensing model between the input electrical parameters of the motor and the polished rod load of the beam pumping unit balanced by the crank.

## 4. Simulation and Optimization of the Soft Sensor for the Indicator Diagram of the Pumping Unit

Combined with the measured input electrical parameters of the motor, the inclination angle of the walking beam, and the actual parameters of the four-bar linkage and the balancing device of the pumping unit, the mathematical model of the polished rod displacement of the pumping unit and the relationship model between the polished rod load and the motor input electrical parameters were simulated, analyzed, and optimized using MATLAB 2019a.

The simulation calculation was only carried out with the example of Well Wang 11–252 in the Wangyao operation area of Changqing Oilfield No. 1 Oil Production Plant and the supporting CYJY5–1.8–13HB pumping unit. As the geometric dimensions of the pumping unit have a huge influence on the calculation of the torque coefficient, the geometric dimensions of the pumping unit were measured and verified. The data after the actual verification and the basic parameters of the CYJY5–1.8–13HB pumping unit are shown in Table 1 below.

**Figure 7:**
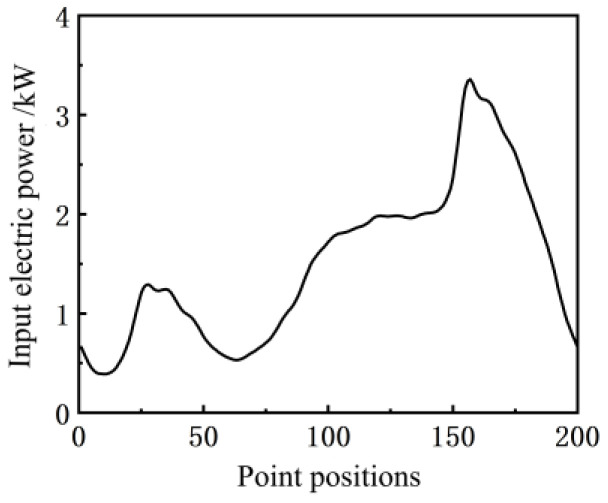
Electric power curve.

**Figure 8:**
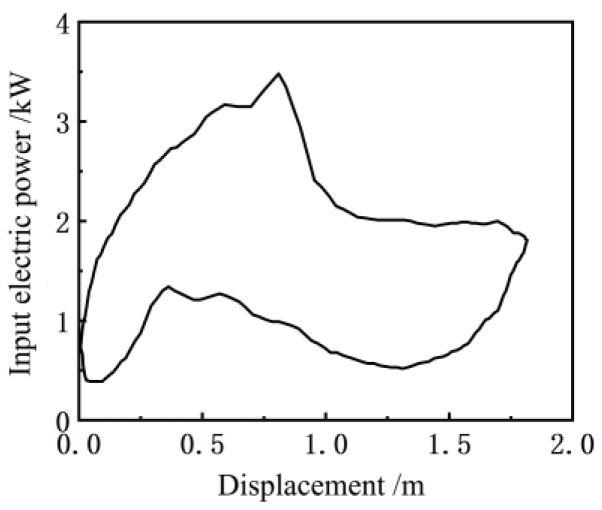
Electric power diagram.

When collecting the dip angle of the walking beam on the pumping well, the positive sequence electric power curve of the input motor synchronously measured by the electric parameter acquisition terminal is shown in Figure 7, and the electric power diagram curve formed with the suspension point displacement is shown in Figure 8.

**Figure 9:**
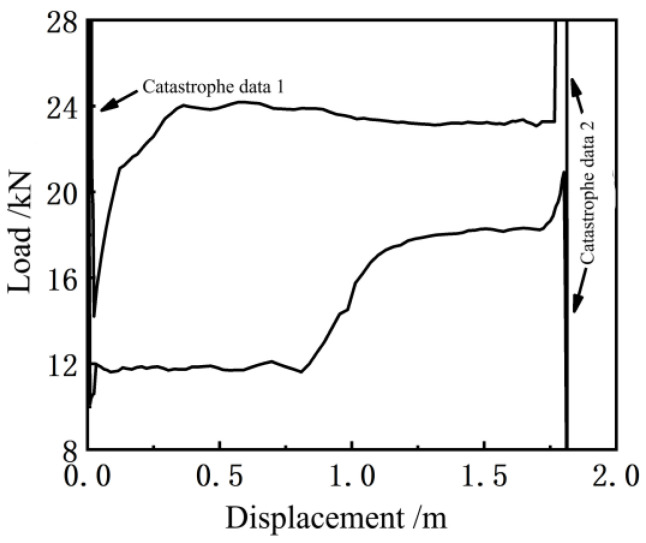
Soft sensor indicator diagram curve.

The input electrical parameters of the motor and the crank angle measured in real-time were substituted into Equations (2) and (25) to calculate the polished rod displacement and the load of the pumping unit. The indicator diagram is shown in Figure 9. (The *x*-axis represents the polished rod load, in kN; The *y*-axis represents the polished rod displacement, in meters).

The indicator diagram obtained by this measurement method often has individual ‘catastrophe data’ near the top and bottom dead points, as indicated in Figure 9 by 1 and 2. Among these ‘catastrophe data’, some are caused by the reverse acceleration of the suspension point and the small displacement of the polished rod due to the failure of the oil well or the working condition of the pump, whereas the load has a sudden change [23]. Some are caused by the singular point near the top and bottom dead points, where a torque factor close to zero is involved in the calculation of the numerical ratio (in Equation (25), the torque factor is the denominator), which is also called the singular change. When drawing the indicator diagram of the pumping unit, it is necessary to identify the nature of these sudden changes and eliminate the singular sudden changes to ensure the accuracy of the indicator diagram measurement and achieve better analysis results of the working conditions. In this study, the singular mutation caused by the calculation of the mathematical ratio has been judged and removed with the help of the La Yida data selection standard [24]. The specific steps of the method are as follows:

(1) Calculate the average value, ${\bar{x}}_{up}$, and the residual error, $v_{i}=x_{i}-{\bar{x}}_{up}$, of the up-stroke load data.
(2) Calculate the standard deviation, $\sigma_{up}$, of the load data of the up-stroke by the Bessel formula.
(3) Compare $x_{i}-\bar{x}$ with $3\sigma_{up}$. If $\left|x_{i}-\bar{x}\right|>3\sigma_{up}$, then the singular mutation value should be discarded. Otherwise, it should be retained.
(4) The discarded values are supplemented by multiple imputations.
(5) The data processing method of the down-stroke load is the same as that for the up-stroke load.

**Figure 10:**
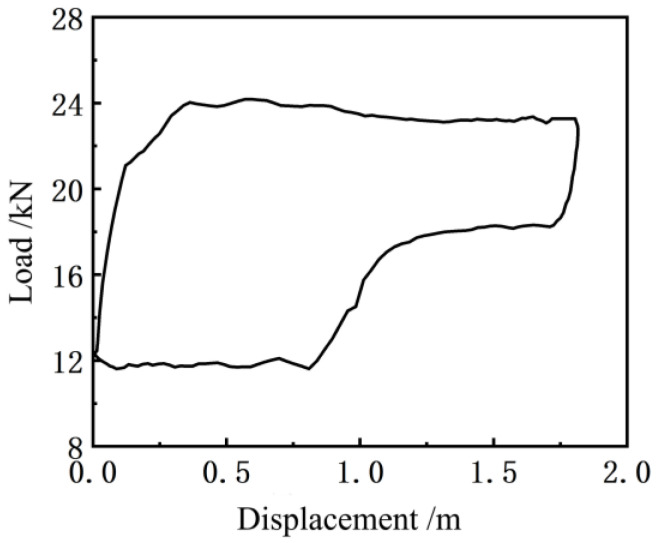
Soft sensor indicator diagram after removing the singular mutation value.

The indicator diagram obtained by judging and removing the singular mutation value in Figure 9 using the La Yida data selection standard is shown in Figure 10.

By comparing Figure 9 and Figure 10, it can be seen that the sudden change in the value caused by the working condition of the pump can be retained by using the La Yida data selection standard, and the singular sudden change in the value caused by the mathematical calculation can be eliminated correctly so that the fault information of the pumping unit system will not be lost and the indicator diagram can be drawn correctly.

**Figure 11:**
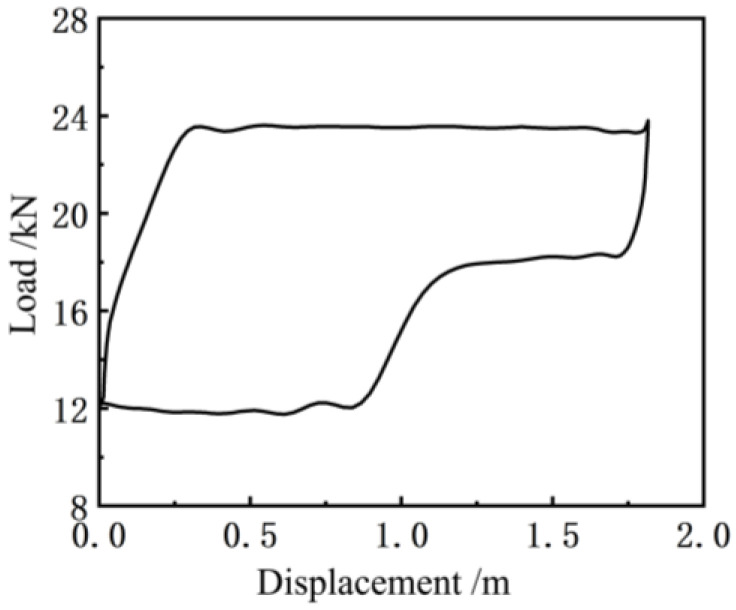
An indicator graph curve measured by the indicator.

**Figure 12:**
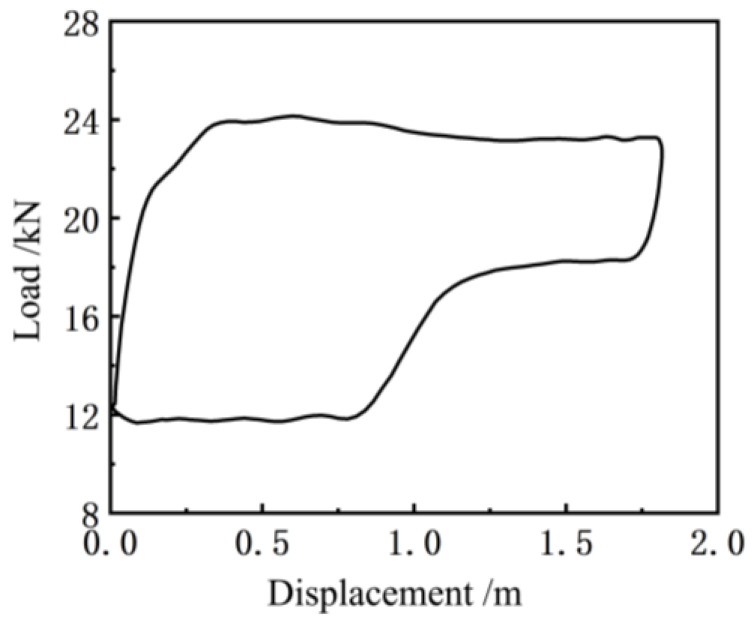
Soft sensor indicator graph curve after filtering.

Figure 11 shows the indicator diagram curve measured by the existing indicator installed at the suspension point of the pumping unit (model: Huakong HK1301, based on the principle of the load-displacement sensor). Before the experiment, the indicator was calibrated in the instrument room of the test team of the First Oil Production Plant of Changqing Oilfield, which ensured that the measurement error of the load and the length is not more than 1%. Compared to Figure 10, it can be seen that the indicator curve obtained by the soft measurement method has more noise interference, and thus it is necessary to smooth the indicator curve obtained by this method [25]. Since the Butterworth filter has a good suppression effect on clutter, a second-order Butterworth filter was applied to Figure 10, and the results thus obtained are shown in Figure 12 below.

Compared to Figure 10, it can be observed that the curve of the soft sensor indicator diagram (Figure 12) after second-order Butterworth filtering is smoother and closer to the actual indicator diagram curve (Figure 11) measured by the existing indicator, which can accurately reflect the change in the trend of the actual indicator diagram curve.

## 5. Field Test of Soft Sensing for the Indicator Diagram of the Pumping Unit

### 5.1. Soft Measurement and Control Platform for the Indicator Diagram of the Beam Pumping Unit

**Figure 13:**
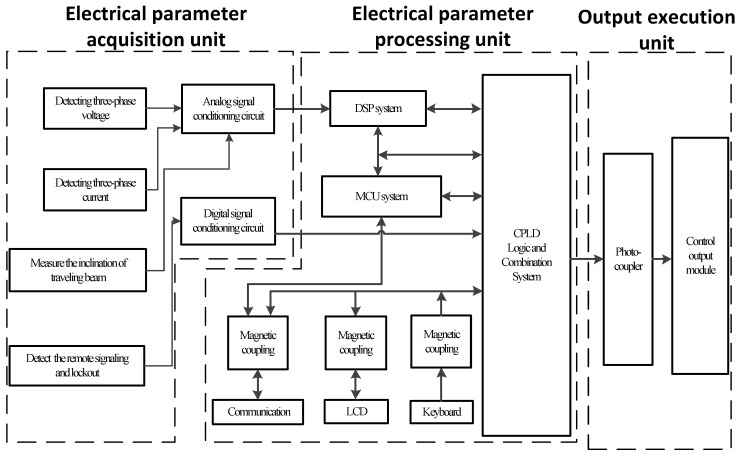
Structure of the intelligent measurement and control platform for the soft-sensing indicator diagram of the beam pumping unit.

The existing intelligent measurement and control platform was used for the soft measurement of the indicator diagram of a beam pumping unit, as shown in Figure 13, to verify the engineering application of the above-established indicator diagram soft measurement model. The hardware structure of the platform includes three parts: the electrical parameter acquisition unit, the electrical parameter processing unit, and the output execution unit.

**Figure 14:**
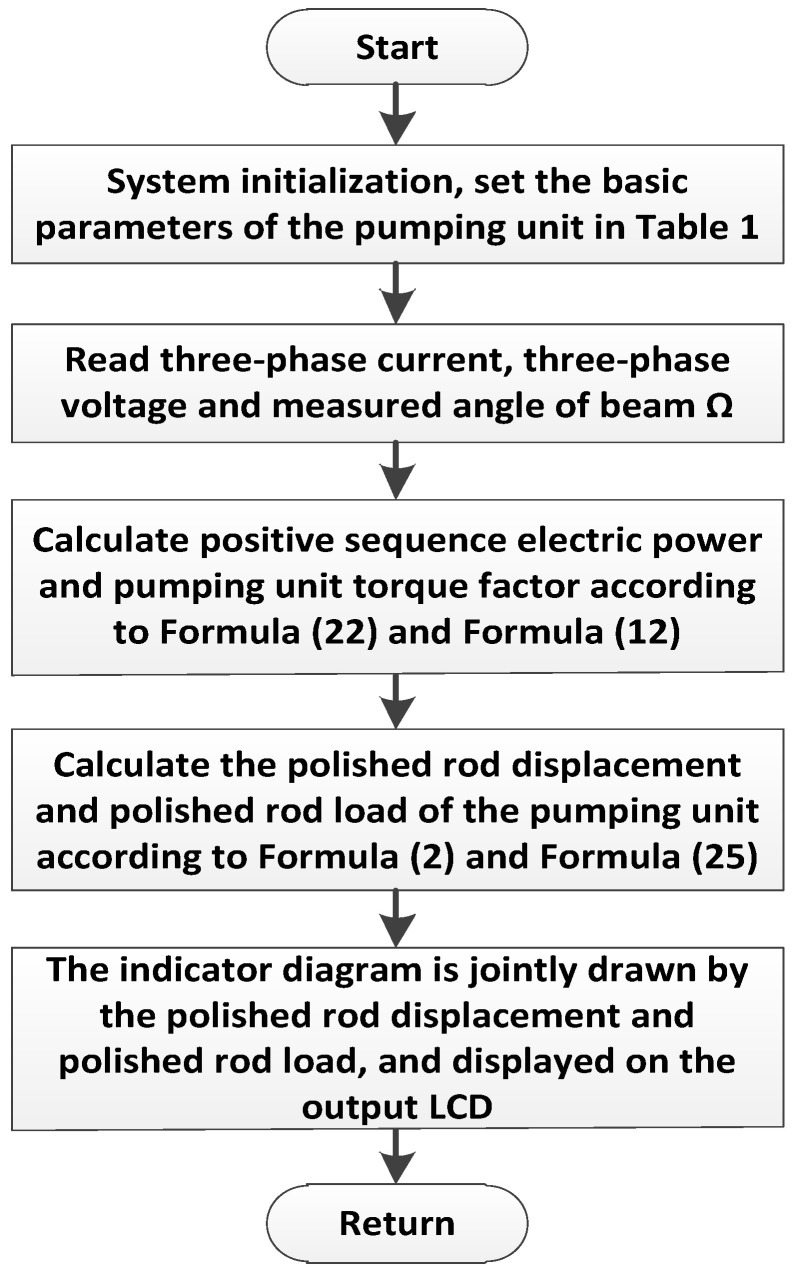
Flowchart of the soft-sensing software for suspension load of the beam pumping unit.

During operation, the electrical parameter acquisition unit collects the three-phase instantaneous voltage ($u_{a}$, $u_{b}$, $u_{c}$) and three-phase instantaneous current ($i_{a}$, $i_{b}$, $i_{c}$) in real time through the voltage and current transformers, collects the angle signal of the traveling beam through the angle sensor of the traveling beam, and transmits it to the electrical parameter processing unit after filtering and digitization by the signal conditioning circuit. The electrical parameter processing unit is composed of a single-chip microcomputer, a Digital signal processor, and a Complex programmable logic device logic and combination unit. It is mainly responsible for calculating the average electrical power of the positive sequence input of the motor, the acquisition of the beam inclination angle, and the calculation of the polished rod displacement and the polished rod load, respectively. It uses the established mathematical model of the horsehead polished rod displacement and torque factor of the pumping unit and the correlation model of the input electrical parameters of the motor, the parameters of the four-bar linkage of the pumping unit, and the polished rod load of the pumping unit. This completes the soft measurement, drawing, and display of the indicator diagram. The output execution unit outputs the control logic. The software flow chart is shown in Figure 14.

### 5.2. Field Test and Analysis of Soft Sensor for the Indicator Diagram of the Beam Pumping Unit

**Figure 15:**
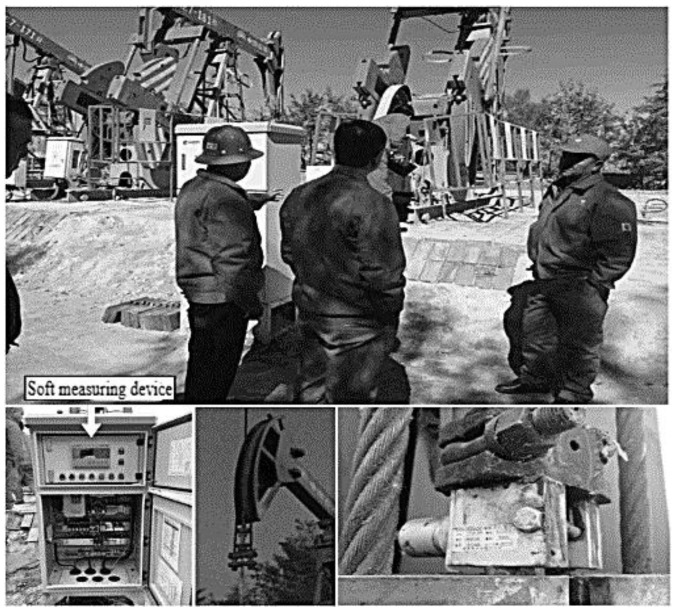
Field test photographs of the oilfield well site.

The soft sensing intelligent measurement and control platform for the indicator diagram of the beam pumping unit was installed in the electrical control cabinet of the pumping unit, and the required signal and control wiring was connected. Field tests and applications were carried out in 30 oil wells randomly selected in the Wangyao operation area of Changqing No. 1 Oil Production Plant. The physical installation diagram is shown in Figure 15.

**Figure 16:**
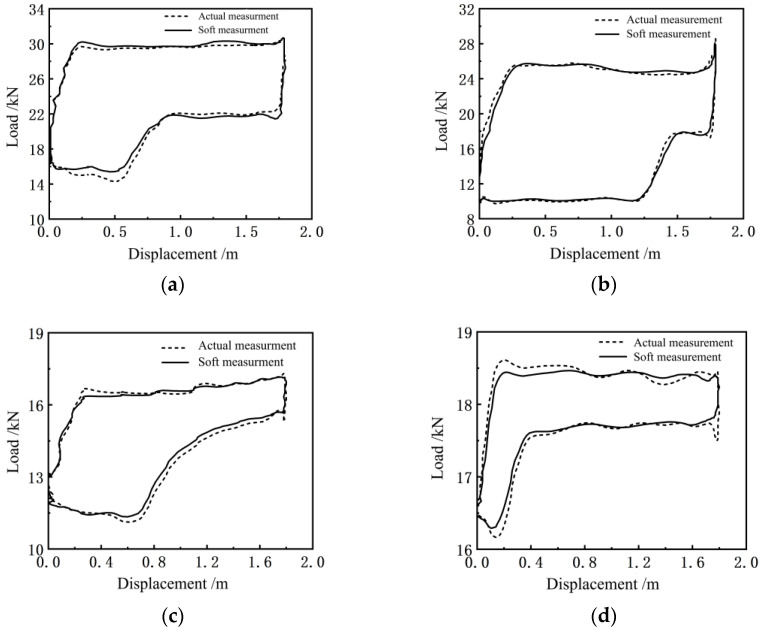

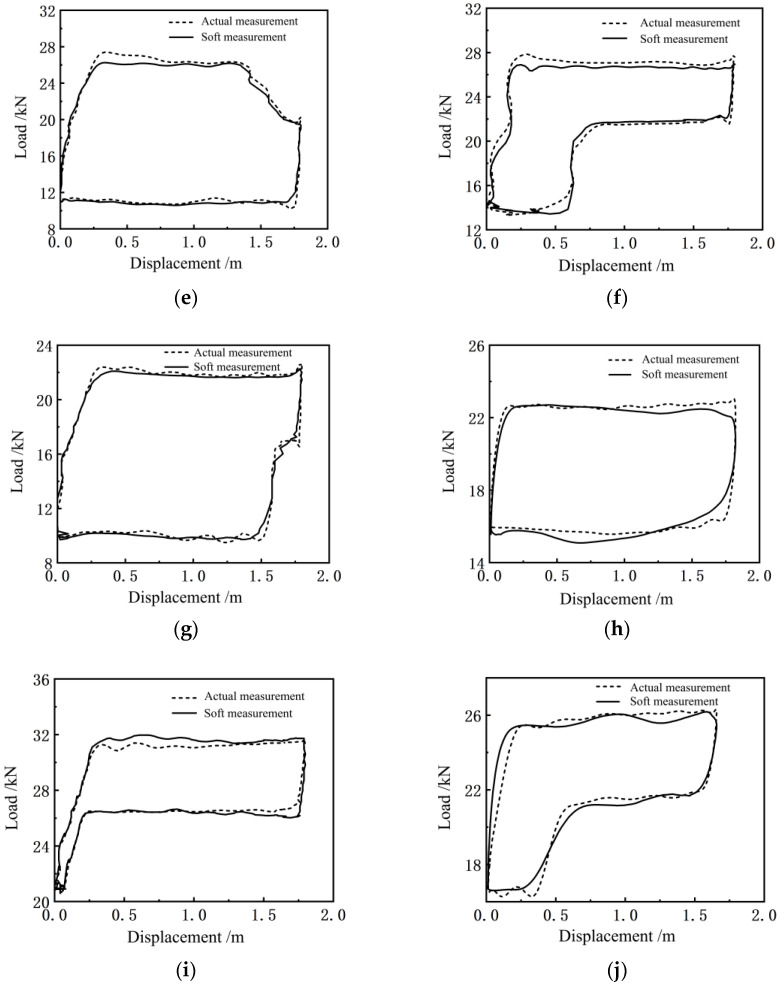
Comparison curves of the indicator diagrams measured by the indicator and the soft sensor of (**a**) Wang 10-252 well, (**b**) Wang 13-282 well, (**c**) Wang 13-251 well, (**d**) Wang 10-024 well, (**e**) Wang 14-241 well, (**f**) Wang 15-26 well, (**g**) Wang 16-241 well, (**h**) Wangbian 15-26 well, (**i**) Wangbian 14-27 well and (**j**) Wangbian 14-28 well.

Figure 16a–j show the comparison results between the measured indicator diagrams of the existing indicators of 10 typical oil wells and the indicator diagram of the soft sensing method presented in this paper (The dotted line represents measured indicator diagram and the solid line represents the indicator diagram of the soft sensing method). These 10 oil wells have been selected from different installation sites, different types of pumping units, and motors. The measurement data of the indicator diagrams of the pumping units of the remaining 20 oil wells is not presented in this paper, since their measurement errors are less than those of the 10 selected oil wells, which does not affect the engineering application conclusions drawn from this study.

It can be seen from Figure 16 that there is a small error between the measured indicator diagram curve of the soft sensing method and the measured indicator diagram curve of the existing indicator, which reflects the trend of the indicator diagram, and can be trusted and adopted for the condition analysis and fault diagnosis of pumping units and deep-well pumps.

**Figure 17:**
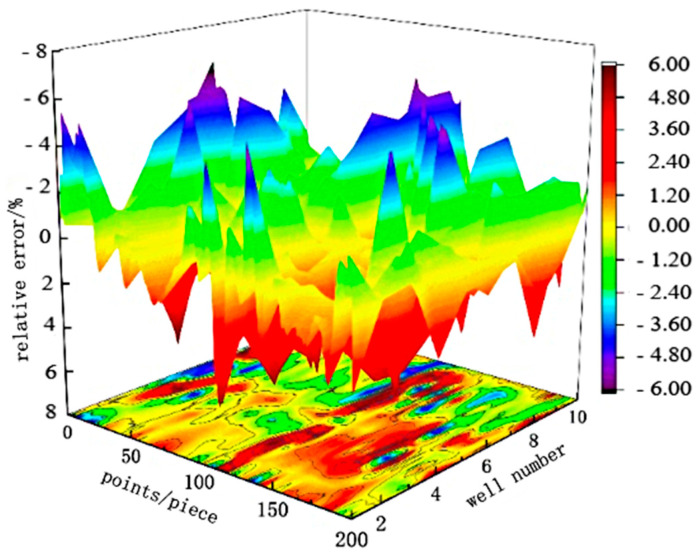
Relative error distribution diagram of 2000 point load data of 10 oil wells with the same displacement.

In order to further analyze the actual measurement effect of the indicator diagram soft measurement method proposed in this work quantitatively, under the same displacement measurement conditions, for each of the selected 10 typical oil wells, the soft measurement method, and the existing indicator were used, respectively, to collect 200 data samples of the polished rod load in a stroke cycle for one-to-one comparison. The specific data and the relative error are shown in Figure 17. The maximum point of relative error between the measured load and the calculated load is given in Table 2.

From Table 2 and Figure 17, it can be seen that under the same displacement measurement conditions, the average relative error of the polished rod load measured by the soft sensor method and the dynamometer method is 3.95%. Yin’s model with crank angle as input has an error rate of 10% [14]. Lu’s model for solving indicator diagram based on FOA-BP neural network optimization has an average relative error of 4.82% [15].

**Figure 18:**
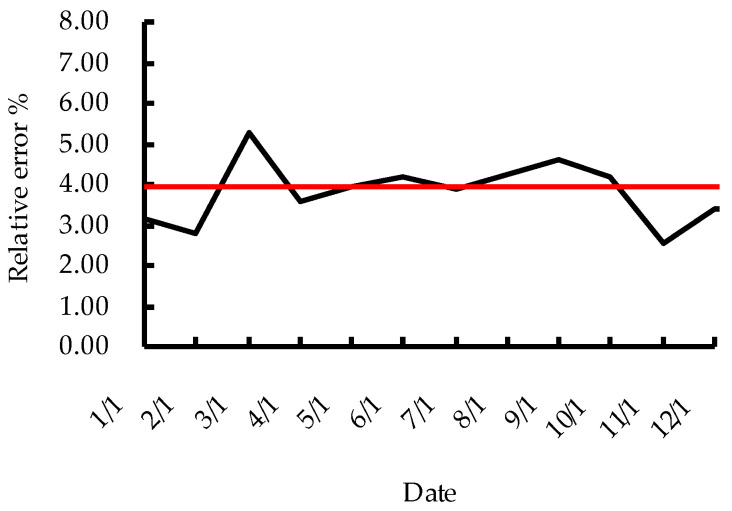
Maximum relative error of well Wang 14-241 and average relative error of 10 oil wells.

In order to verify the stability of the method described in this paper, well Wang 14-241, whose maximum relative error is close to the average relative error of 10 oil wells, is selected as the monitoring oil well, and the polished rod load data of the pumping unit of this oil well is collected by the existing dynamometer every other month in 2023 and compared with the polished rod load data collected by the soft sensing method. The maximum relative error data of the measured load and the soft sensing load are shown in Table 3, The comparison with the average relative error obtained from 10 oil wells is shown in Figure 18.

It can be seen from Table 3 and Figure 18 that the maximum relative error (shown by the black) of the polished rod load obtained by this soft sensing method during the continuous operation of well Wang 14-241 for one year remains within the range of plus or minus 2% of the average relative error (shown by the red) obtained from 10 typical wells.

The above engineering applications and experimental results prove that this indicator diagram soft measurement method does not have the problem of poor stability that is encountered in the conventional load-displacement sensor method due to the impact of an alternating load on its force sensor. In addition, it is not necessary to measure the crank angle. The method proposed in this work is highly practical in engineering applications, and its accuracy also meets the requirements of the oil field.

## 6. Conclusions

In this work, a new mathematical model of polished rod displacement and torque factor has been established by directly replacing the conventional crank angle with the easily measured beam inclination angle, which can accurately and conveniently realize the engineering measurement of the polished rod displacement and torque factor.

By analyzing the motion law of the beam pumping unit system, the correlation between the input electrical parameters of the motor, the parameters of the four-bar linkage, and the polished rod load has been established, which can realize the measurement of the polished rod load, and then draw the indicator diagram online with the displacement. The results of the simulation test and application verification in many oil wells on the oil field show that the indicator diagram soft measurement method proposed in this study can achieve online accurate measurement of the indicator diagram, has good stability, strong engineering application, and the average relative error is not more than 3.95%; thus, meeting the production requirements of the project site.

The method described in this paper only studies the beam pumping units with the most widely used crank balance mode. For beam pumping units with beam balance and compound balance and other types of pumping units, the relevant indicator diagram soft sensing model can be established according to the idea of this paper, combined with the kinematics law and force analysis. The method described in this paper can be transferred to the application of oilfield fault diagnosis and other technologies based on indicator diagram, and also provides a new perspective based on soft sensing technology for the related fields with electric energy as the power source to explore its internal hidden unknown mechanism.

## Figures and Tables

**Table 1:** Basic parameters of the CYJY5–1.8–13HB pumping unit.

Parameter Type	Parameter Value
*A* (/m)	1.720
*C* (/m)	1.520
*P* (/m)	2.666
*R* (/m)	0.677
*K* (/m)	3.400
*H* (/m)	2.540
*I* (/m)	2.490
*I_b_* (/m)	2.530
*n* (/min^−1^)	3.4
*η* (/%)	90
*η_E_* (/%)	85
*B* (/kN)	−0.3
$Q_{e}$ (/kN)	4

**Table 2:** Maximum relative error of the suspension load measured by the dynamometer and the soft sensor.

Well Number	Displacement (m)	Calculated Load (kN)	Measureed Load (kN)	Maximum Relative Error (%)
Wang 10-252	0.50	15.29	14.43	5.95
Wang 13-282	1.75	18.25	17.46	4.52
Wang 13-251	1.77	17.88	17.51	2.11
Wang 10-024	0.27	16.36	16.68	−1.91
Wang 14-241	1.72	11.05	10.67	3.56
Wang 15-26	0.33	26.34	27.71	−4.94
Wang 16-241	1.49	10.35	9.84	5.18
Wangbian 15-26	1.72	17.26	16.53	4.41
Wangbian 14-27	0.45	31.62	30.84	2.52
Wangbian 14-28	0.32	17.03	16.32	4.35
Average relative error	3.95%			

**Table 3:** Maximum relative error between measured load and soft sensing load of well Wang 14-241.

Date (2023)	Maximum Relative Error (%)	Date (2023)	Maximum Relative Error (%)	Date (2023)	Maximum Relative Error (%)
1/1	3.16%	5/1	3.91%	9/1	4.62%
2/1	2.76%	6/1	4.19%	10/1	4.18%
3/1	5.28%	7/1	3.87%	11/1	2.55%
4/1	3.56%	8/1	3.36%	12/1	3.41%

## Data Availability

Data sharing is not applicable to this article as no datasets were generated during the current study.

## References

[1] Xiong W. (2020). Discussion on the application of indicator diagram in oil field energy conservation monitoring. Energy Conserv. Petrol. Petrochem. Ind..

[2] Liu H.Y. (2021). Application strategy of Indicator Diagram technology in oilfield. Chem. Eng. Equip..

[3] Tian H., Deng S., Wang C.B., Ni X.Y., Wang H., Liu Y. (2021). A novel method for prediction of paraffin deposit in sucker rod pumping system based on CNN indicator diagram feature deep learning. J. Petrol. Sci. Eng..

[4] Liu C.Q., Zhu H.Q. (2020). Research of Integrated Wireless Dynamometer for Oil Well Measurement. Comput. Digit. Eng..

[5] Tian H.F., Yu X.C. (2019). Design of indicator diagram sensing system based on position sensing and displacement multiplexing. J. Instrum..

[6] Gibbs S.G., Neely A.B. (1966). Computer diagnosis of down-hole conditions in sucker rod pumping wells. J. Petrol. Technol..

[7] Doty D.R., Schmidt Z. (1983). An improved model for sucker rod pumping. Soc. Petrol. Eng. J..

[8] Peng Y., Yan W.H. (2001). A Quick Recursion Arithmetic of Calculating Pump Dynamograph of the Sucker-rod Pumping System. Drill. Prod. Technol..

[9] Li H.J., Li Y., Long Q.S. (1992). Calculation of dynamometer diagram of the pre pumping well with measured power curve. Oil Field Equip..

[10] Lv Z.Z., Jiang C., Jiang L., Zhong G.X. (2019). Study on the test system of oil pumping machine diagram based on electric power. Mach. Des. Manuf..

[11] Li X.Y., Gao X.W., Yuan C.H., Hou Y.B. (2020). Mechanism modeling of multiphase fluid pumping process. J. Mech. Eng..

[12] Zhang Y., Qiu Z., Zhao H., Zhu L. Power integration based dynamic equilibrium identification method of beam pumping system. Proceedings of the 2016 Annual IEEE Systems Conference (SysCon).

[13] Wang Y. (2018). Research on Energy Consumption Characteristics and Key Technologies of Motor System under Periodic Potential Energy Load.

[14] Yin X.J., Du Z.L., Wang Y.X. (2022). Analysis and experimental study of oil well indicator diagram based on electric parameter method. Energy Rep..

[15] Lu Y. (2023). Hanging point indicator diagram inversion technology based on FOA-BP neural network. Oil Gas Field Surf. Eng..

[16] Tan L., Xie Q., Wang G.M. (2021). Research on pumping unit indicator diagram test system based on electric power. Equip. Manag. Maint..

[17] Yu H.X., Hu J.T. (2011). Speed and load torque estimation of induction motor using extended Kalman filter, Chin. J. Sci. Instrum..

[18] Zhang J.Z., Li X.Q., Shi H.N. (2005). Design Calculation of Beam Pumping Unit.

[19] Huang H. (2022). Application of symmetric component method in solving asymmetric short-circuit current of power transformer. China Electr. Eng..

[20] Mao Z.J., Li J. (2023). Research on Calculation Methods for Asymmetric Operation of Power Systems. J. Xingyi Ethn. Norm. Univ..

[21] Zhao H.J., Zhang Y., Zhu L.J., Qiu Z.M. Power integration based dynamic equilibrium measurement and control device of beam pumping unit. Proceedings of the 2016 IEEE International Instrumentation and Measurement Technology Conference Proceedings.

[22] Zhan G.B., Zong Z.H., Ji Z. (2020). Torque control of permanent magnet synchronous motor using extended Kalman filter. Micromotor.

[23] Dong S.M., Li W.C., Hou T.B., Wang H.B., Chen J.X. (2016). Optimizing the Running Par ameters of a Variable Frequency Beam Pumping System and Simulating Its Dynamic Behaviors. J. Mech. Eng..

[24] Liu X.H. (2021). Fault identification of digital weighing sensor based on statistical analysis method. China Instrum. Meter..

[25] Yang G., Wang T.W., Zhang P. (2020). Research on curve smoothing algorithm for diesel indicator diagram. Vibroeng. Procedia.

